# Self-Compassion and Adherence in Five Medical Samples: the Role of Stress

**DOI:** 10.1007/s12671-018-0945-9

**Published:** 2018-04-10

**Authors:** Fuschia M. Sirois, Jameson K. Hirsch

**Affiliations:** 1 Department of Psychology, University of Sheffield, Cathedral Court, 1 Vicar Lane, Sheffield, S1 2LT UK; 2 Department of Psychology, East Tennessee State University, Johnson City, TN USA

**Keywords:** Self-compassion, Adherence, Stress, Chronic illness, Health behaviours

## Abstract

Emerging evidence indicates self-compassion can be beneficial for medical populations and for medical adherence; yet, research to date has not fully examined the reasons for this association. This study examined the association of dispositional self-compassion to adherence across five medical samples and tested the extent to which perceived stress accounted for this association. Five medical samples (total *N* = 709), including fibromyalgia, chronic fatigue syndrome, and cancer patients, recruited from various sources, completed online surveys. Self-compassion was positively associated with adherence in all five samples. A meta-analysis of the associations revealed a small average effect size (average *r* = .22, [0.15, 0.29]) of self-compassion and adherence and non-significant heterogeneity among the effects (*Q* (4) = 3.15, *p* = .532). A meta-analysis of the kappa^2^ values from the indirect effects of self-compassion on adherence revealed that, on average, 11% of the variance in medical adherence that was explained by self-compassion could be attributed to lower perceived stress. Overall, findings demonstrate that dispositional self-compassion is associated with better medical adherence among people with fibromyalgia, chronic fatigue syndrome, and cancer, due in part to lower stress. This research contributes to a growing evidence base indicating the value of self-compassion for health-related behaviours in a variety of medical populations.

## Introduction

A burgeoning body of research indicates that self-compassion, taking a kind, accepting, and mindful stance towards oneself when dealing with challenges and failures (Neff 2003b), can be beneficial for a range of health-related outcomes, including the practice of important health behaviours. For example, several studies have now demonstrated that in healthy populations, self-compassion is associated with better exercise behaviours (Magnus et al. 2010), healthy eating behaviours (Adams and Leary 2007; Schoenefeld and Webb 2013), weight management (Mantzios and Wilson 2015), smoking cessation (Kelly et al. 2010), seeking medical care (Terry et al. 2013), and measures of general health-promoting behaviours including healthy eating, adequate exercise, restful sleep, and avoiding unhealthy foods (Dunne et al. 2016; Homan and Sirois 2017; Sirois et al. 2015a). However, for individuals living with long-term conditions that require ongoing medical care and monitoring, engaging in health-promoting behaviours are especially important for the maintenance of health. Accordingly, it has been noted that self-compassion could be particularly beneficial for promoting the self-regulation of health behaviours in the context of chronic health conditions (Sirois and Rowse 2016).

Consonant with this view, self-compassion may be beneficial for facilitating patient adherence to medical recommendations. Defined as the extent to which an individual engages in health recommendations from a health-care provider (Taylor and Sirois 2014), patient adherence is a health behaviour with important implications for a variety of patient contexts and outcomes. Poor patient adherence is associated with costs in the 100s of billions of dollars annually in the USA (Martin et al. 2005) and is a key contributor to morbidity and mortality (Martin et al. 2005). Consistent adherence, in contrast, is linked to improved physical health outcomes, lower medication use, and better health-related quality of life (DiMatteo 2004; Dobkin et al. 2009). More generally, patient adherence can include the following health-care provider-recommended regimes such as getting regular diagnostic tests and check-ups, taking medication, and making lifestyle changes involving diet and exercise changes necessary for the maintenance of health status (Taylor and Sirois 2014). However, adherence to recommendations for lifestyle changes tends to be particularly challenging for patients and can be as low as 19% (Martin et al. 2005).

Together, the three dimensions of self-compassion—self-kindness (versus self-judgement), common humanity (versus isolation), and mindfulness (versus over-identification) (Neff 2003b)—are theorised to bolster the self-regulatory resources necessary for engaging in self-care behaviours, including monitoring and evaluating ongoing behaviour, and emotional regulation (Sirois 2015b; Terry and Leary 2011). For example, self-compassion is proposed to facilitate adaptive emotional responding to the inevitable setbacks that occur when trying to change health behaviours (Sirois et al. 2015a), by promoting self-kind versus self-blaming responses, and mindful acceptance of medical challenges (Brion et al. 2014; Friis et al. 2015a). Accordingly, self-compassionate people may be less depleted by their medical conditions and thus have more energy to direct towards effective self-regulation of health behaviours (Terry and Leary 2011). Self-compassion has also been posited to provide emotional stability that fosters taking responsibility for one’s health, despite the initial discomfort associated with making changes to important health behaviours, such as exercise and diet (Friis et al. 2015a). In these respects, self-compassion has the potential to be an important resource for promoting patient adherence.

Emerging evidence supports this view. In a study of HIV patients, trait self-compassion was associated with better adherence to following HIV treatments, keeping appointments, and taking medications on time (Brion et al. 2014). Importantly, lower feeling of shame associated with high self-compassion was noted as an explanatory factor among the group of patients more likely to adhere. In a more rigorous test of the links between self-compassion and self-care adherence, a randomised controlled trial of an 8-week group-based mindful self-compassion intervention on distress and self-care in diabetes patients found that, compared to the waitlist control group, those in the intervention group reported significantly lower depression and diabetes distress, as well as a statistically meaningful decrease in HbA1c (an indicator of glycemic control) at the 3-month follow-up (Friis et al. 2016). Similarly, Dowd and Jung (2017) examined self-compassion at baseline in relation to dietary adherence 1 month later in a sample of adults with celiac disease. For individuals with celiac disease, strict adherence to a gluten-free diet is the only known treatment for managing this condition. Although the results indicated that self-compassion indirectly predicted stricter adherence to a gluten-free diet at time 2 via self-regulatory efficacy, the direct path remained significant, suggesting that other factors may account for why self-compassion promotes adherence.

One factor associated with (low) self-compassion, and known to be disruptive to patient adherence (Bottonari et al. 2010), and the self-regulation of health behaviours more generally (Burg et al. 2017; Rod et al. 2009; Sirois 2007), is perceived stress. The links between self-compassion and lower stress have been demonstrated using both subjective and objective measures of stress. Trait self-compassion is associated with lower self-reported stress for several patient groups including diabetes (Friis et al. 2015b), arthritis and inflammatory bowel disease (Sirois et al. 2015b), cancer and mixed chronic illnesses (Pinto-Gouveia et al. 2014), breast cancer (Przezdziecki et al. 2013), and HIV (Brion et al. 2014). The directionality of the association between self-compassion and stress assumed in this research is further supported by evidence from several studies. For example, among individuals exposed to laboratory task inducing psychosocial stress, those high in self-compassion had lower levels of the pro-inflammatory cytokine, interleukin-6, in response to the stressor (Breines et al. 2014). In another study, women who had brief training in self-compassion in comparison to control groups had lower levels of salivary alpha-amylase, a marker of sympathetic nervous system reactivity, and more adaptive heart rate variability, a marker of healthy parasympathetic nervous system reactivity, in response to a stress-inducing lab task (Arch et al. 2014).

When considered as a negative affective state, stress is theorised to derail self-regulation efforts by monopolising important self-regulation resources such as temporal focus (Sirois 2016), and by interfering with the self-monitoring necessary for enacting health behaviour goals (Wagner and Heatherton 2015). For individuals who are already dealing with the daily challenges of stressful symptoms that can accompany living with a chronic health condition, or who are fatigued from their condition, stress may be particularly detrimental for keeping up with important self-care activities such as following medical regimens and recommended lifestyle changes (Jerant et al. 2005; Rod et al. 2009; Sirois 2015a). Under such conditions, failed attempts to follow through and adhere to recommendations can trigger stress-generating self-critical and self-blaming responses for not being able to meet personal and others’ expectations about adequately engaging in self-care or managing one’s condition (Moskovitz et al. 2000; Voth and Sirois 2009). Such responses can add to further depletion of the energy and focus required to effectively manage one’s health condition.

Self-compassion includes a focus on mindful acceptance of personal failure and maintaining emotional balance rather than over-identifying with the negative feelings that arise from personal failures (Neff 2003b), such as lapses in self-care and disease management. In this respect, self-compassion may reduce not only the stress of the daily challenges of living with a long-term health condition, but also the collateral stress that can arise when attempts at self-care and following medical regimens fail (Sirois and Rowse 2016).

The aim of the present study was to replicate and extend previous work demonstrating that self-compassion is associated with better adherence in medical populations, by testing the association of dispositional self-compassion to adherence across five medical samples consisting of three chronic health conditions: fibromyalgia, chronic fatigue syndrome, and cancer. Each of these conditions has fatigue as a common symptom (Carlson et al. 2004; Clayton 2015; Wolfe et al. 2010), which can interfere with illness self-management (Jerant et al. 2005). Consistent with theory and research noting that self-compassion is linked to lower stress in medical samples (Friis et al. 2015b; Sirois et al. 2015b) and that lower stress is associated with better adherence (Bottonari et al. 2010), we also tested the extent to which perceived stress accounted for the association of self-compassion with adherence. Specifically, we hypothesised that self-compassion would be associated with better general medical adherence in the five medical samples and that lower stress would account for a significant proportion of the variance in this association. To summarise the overall associations of self-compassion with adherence via stress, we statistically meta-analysed the bivariate and multivariate effects and estimate their sizes. Because previous research has noted that women tend to have lower levels of self-compassion compared to men (Neff 2011) and that women tend to be less adherent to some medical regimens than men (Manteuffel et al. 2013), we also tested the potential moderating role of participant sex in the proposed associations among self-compassion, stress, and medical adherence. Age was also tested as a moderator as there is mixed evidence regarding its effects on adherence (DiMatteo 2004).

## Method

### Participants

The current study included data from five independent samples (total *N* = 709) of people with medical conditions (two samples with fibromyalgia syndrome (FMS), two samples with cancer, and one sample with CFS) collected from 2010 to 2016 in the United States, Canada, and the United Kingdom, as part of the authors’ larger research program on the role of self-compassion in health behaviours and outcomes. Samples 1 and 2 consisted of patients who self-reported being medically diagnosed with FMS. Sample 3 (*n* = 61) consisted of patients who self-reported being medically diagnosed with CFS. Sample 4 (*n* = 55) consisted of patients with cancer and sample 5 (*n* = 122) consisted of patients in remission from cancer. For all samples, list-wise deletion was used to remove cases with missing values on the study variables prior to analyses. The demographic characteristics of the five medical samples are presented in Table 1. For the most part, the samples were predominantly White and female.

**Table 1:** Demographic characteristics of the five medical samples

				Age (years)		Education level (%)		
Sample	*N*	Percent female	Percent white	*M*	SD	High school	College/university	Graduate school
1. Fibromyalgia 1	319	96.1	92.7	47.89	12.7	35.8	47.6	16.7
2. Fibromyalgia 2	152	89.4	86.3	41.51	14.02	18.5	63.6	17.9
3. Chronic Fatigue syndrome	61	83.8	93.8	33.91	14.8	11.8	58.8	29.5
4. Cancer	55	62.0	100.00	61.24	11.24	9.0	58.2	31.4
5. Cancer survivor	122	64.4	100.00	61.47	12.39	7.4	67.3	24.1

### Procedure

Ethical clearance for the data collection was obtained from the authors’ Institutional Review Boards prior to recruitment and data collection. Sample 1 (*n* = 319) was recruited via advertisement and contact with local, regional, and national psychoeducational FMS organizations and support groups. Sample 2 (*n* = 152) was recruited via a university volunteers list and social media. Sample 3 was recruited in the same manner as sample 2. Participants in samples 4 and 5 were recruited via advertisements distributed through national cancer organizations in the USA and through social media. All samples completed their respective surveys online in exchange for a chance to win gift vouchers of varying values. Links to relevant mental health resources were provided to all participants upon completion of the survey.

### Measures

In addition to demographic questions, all samples completed measures of the following constructs using measures available from the data sets. Descriptive data for all measures are presented in Table 2.

**Table 2:** Descriptive statistics for the measures used in the five medical samples

	Self-compassion (SC)		Medical adherence (MOS-GA)		Stress (ST)			
Sample	Mean (SD)	Cronbach’s alpha	Mean (SD)	Cronbach’s alpha	Mean (SD)	Cronbach’s alpha	SC-ST (*r*)	ST-MOS-GA (*r*)
1. Fibromyalgia 1	2.91 (.88)	0.88	4.09 (1.08)	0.87	14.93 (4.65)	0.85	− .583**	− .166*
2. Fibromyalgia 2	2.87 (.81)	0.91	3.88 (1.52)	0.94	2.97 (.87)	0.89	− .601**	− .254**
3. Chronic fatigue syndrome	2.78 (.76)	0.76	3.99 (1.46)	0.97	3.36 (.65)	0.85	− .628**	− .395**
4. Cancer	3.39 (.76)	0.87	4.84 (.92)	0.71	2.45 (.70)	0.78	− .625**	− .322**
5. Cancer remission	3.56 (.73)	0.85	4.99 (.83)	0.84	1.95 (.74)	0.81	− .668**	− .330**

*SD* standard deviation

*p < .05, **p < .01

#### Patient Adherence

All samples completed the Medical Outcomes Study Measure of Patient Adherence—General Adherence Items (MOS-GA; Sherbourne et al. 1992). The five-item measure assesses general adherence to medical treatment recommendations on a 6-point Likert scale ranging from “none of the time” to “all of the time”. The MOS-GA has demonstrated good internal consistency in chronic illness samples (Cronbach’s alpha = 0.81) (Sherbourne et al. 1992).

#### Self-Compassion

The short 12-item version of the Self-Compassion Scale (SCS-12; Raes et al. 2011) was completed by all samples. The short version was chosen to minimise participant burden as it was administered as part of a larger questionnaire. Like the full 26-item version (Neff 2003a), the SCS-12 assesses the three main components of self-compassion and their negative counterparts, self-kindness (self-judgement), common humanity (isolation), and mindfulness (over-identification) with both positively and negatively worded items. Respondents indicate how often they behave in the described way using response options ranging from 1 (Almost Never) to 5 (Almost Always). Higher values on the total self-compassion score reflect higher levels of dispositional self-compassion. The SCS-12 has demonstrated good internal consistency in previous research (Cronbach’s alpha = 0.87; Raes et al. 2011).

#### Stress

The fibromyalgia sample 2 and the CFS sample each completed a ten-item version of the Perceived Stress Scale (PSS; Cohen and Williamson 1988), and the cancer patients and survivor samples each completed a four-item version of the PSS, a widely used, empirically established index of general perceived stress. The stressfulness of events within the past month is assessed with items rated on a 5-point scale with response options ranging from “never” to very “often”. Both the PSS-4 and PSS-10 have demonstrated adequate psychometric properties in community samples, with Cronbach’s alphas of 0.60 and 0.78, respectively (Cohen and Williamson 1988).

The fibromyalgia sample 1 completed the Depression Anxiety Stress Scales-21 (DASS-21; Lovibond and Lovibond 1995), which includes a seven-item stress subscale designed to assess difficulty relaxing, nervous arousal, irritability, and impatience. Stress over the past week (e.g. “I found it hard to wind down”; “I found myself getting agitated”) is rated on a 4-point Likert scale ranging from “Never” (0) to “Always” (3). Scores are summed for each subscale and then doubled to allow comparison with the original DASS-42, with a resultant range of 0 to 42 for each subscale. The DASS-21 stress subscale has been successfully used, with adequate internal consistency, in a non-clinical sample (Cronbach’s alpha = 0.88) as well as in pain patients (Cronbach alpha = 0.88) (Henry and Crawford 2005; Wood et al. 2010). As evidence of its validity, the DASS-21 stress subscale is positively related to the PSS (Osman et al. 2012).

### Data Analyses

Bivariate correlations were conducted to assess the raw associations of self-compassion with stress, self-compassion with medical adherence, and stress with medical adherence. The average effect size of the association between self-compassion and medical adherence was estimated from the bivariate correlations with a random effects model meta-analysis conducted with Comprehensive Meta-Analysis (CMA), Version 2 software Borenstein et al. 2005). This software transforms the individual correlation coefficients into Fisher’s *z* scores, weights the effects, and then meta-analyses them.

Two indicators of heterogeneity were chosen to assess whether moderator analyses were warranted. The heterogeneity statistic, *Q*, assesses the degree of variability among the pool of effects sizes (Card 2012), and when significant, moderator analysis is warranted. The *I*^2^ statistic estimated the proportion of variability present that is not due to sampling error within studies. The *I*^2^ value of 25% is evaluated as reflecting low heterogeneity, 50% reflecting moderate heterogeneity, and 75% or more reflecting high heterogeneity (Higgins and Thompson 2002). A mixed effects meta-regression (method of moments) analysis was planned to assess the potential moderating effects of age (recorded as a continuous variable) and sex (recorded as the percentage of the sample that was female) on the average associations of self-compassion with medical adherence across the samples, should the indicators of heterogeneity be significant.

To estimate the extent to which stress explained the variance in the relationship between self-compassion and adherence, the kappa-squared (*k*^2^) statistic was calculated for each sample using the Hayes macro PROCESS (Hayes 2013). The *k*^2^ reflects the proportion of the maximum indirect effect available for explanation by indirect effects that is explained by a mediator (Preacher and Kelley 2011).

#### Data Availability

All data are available upon request from the authors.

## Results

Correlation analysis revealed that across all five samples, self-compassion was negatively associated with stress and positively associated with adherence, and stress was negatively associated with adherence (see Tables 2 and 3). The meta-analysis of the effect sizes across the five samples revealed a small average effect size (average *r* = .22) for the association between self-compassion and adherence.

**Table 3:** Meta-analysed bivariate correlations between self-compassion (SC) and medical adherence (MOS-GA), and kappa^2^ of the indirect effects through stress, across five medical groups (total *N* = 709)

Sample	*N*	SC-MO-SGA (*r*)	95% CI	Kappa^2^	95% CI
1. Fibromyalgia 1	319	.208	[0.10, 0.31]	0.033	[0.00, 0.09]
2. Fibromyalgia 2	152	.130	[− 0.03, 0.28]	0.133	[0.04, 0.24]
3. Chronic fatigue syndrome	61	.314	[0.07, 0.52]	0.201	[0.04, 0.37]
4. Cancer	55	.258	[− 0.01, 0.49]	0.133	[0.01, 0.31]
5. Cancer survivor	122	.311	[0.14, 0.46]	0.116	[0.01, 0.24]
Meta-analysis results	709	.222	[0.15, 0.29]	0.110	[0.04, 0.17]
	*Q* (4)	3.15		7.13	
	*p*	.532		.129	
	*I* ^2^	0.00		43.88	

The two indicators of heterogeneity, the *Q* statistic and the *I*^2^ (Higgins and Thompson 2002), suggested that the variance in effect sizes across the studies was small and non-significant. Accordingly, the planned moderator analyses for age and participant sex were not warranted and therefore not conducted.

The *k*^2^ values were significant for each sample, and the meta-analysis of these effects found that, on average, 11.0% of the variance in medical adherence that was explained by self-compassion could be attributed to lower stress (see Table 3). However, the *Q* statistic and the *I*^2^ indicated a non-significant degree of variability among the effects, and therefore, no moderator analyses were conducted.

## Discussion

Previous research demonstrated a link between self-compassion and health-promoting behaviours (Sirois et al. 2015a), and with adherence to a gluten-free diet (Dowd and Jung 2017), HIV medication (Brion et al. 2014), and glycemic control in diabetes (Friis et al. 2016). The current study replicates and extends this research by finding that dispositional self-compassion was associated with better adherence to physician recommendations across five diverse medical samples, including people with FMS, CFS, and cancer. Importantly, the current study found evidence across all five samples that self-compassion relates to adherence in part due to the lower stress associated with self-compassion. These findings are consistent with a self-regulation view of self-compassion and adherence (Sirois 2015b; Terry and Leary 2011) and provide preliminary support for our proposition that self-compassion is a core resiliency factor that can foster better health behaviours in medical samples by supporting healthy self-care.

Our findings provide promising preliminary evidence to suggest that interventions that focus on increasing self-compassion may be particularly beneficial for enhancing self-care in the context of fibromyalgia and chronic fatigue syndrome, two chronic conditions that are more common among women compared to men (Rusu et al. 2015). Previous evidence demonstrating the effectiveness of self-compassion training for women supports this proposition. After only two 10-min self-compassion training sessions delivered via online recording, 4 days apart, women exposed to a social stress test had lower sympathetic nervous system reactivity (salivary alpha-amylase) and more adaptive parasympathetic nervous system reactivity (heart rate variability) compared to the placebo control and no training control groups (Arch et al. 2014). Although the sample included healthy young women, research with predominantly female samples of people with arthritis and inflammatory bowel disease has also found that dispositional self-compassion had both direct and indirect linkages to lower perceived stress (Sirois et al. 2015b). Taken together with longitudinal research demonstrating that chronic stress prospectively predicted decreases in treatment adherence among individuals with HIV (Bottonari et al. 2010), these findings indicate that further work is needed to understand the potential of self-compassion training for improving adherence among women with medical conditions.

A unique feature of the current study is the inclusion of data from five medical samples, which meant that the results reflect how self-compassion relates to adherence across diverse medical needs. This suggests that the previous findings with respect to the benefits of self-compassion for adherence in HIV, celiac disease, and diabetes patients (Arch et al. 2014; Bottonari et al. 2010; Friis et al. 2016) may also extend to other medical populations and adherence behaviours. The measure of general adherence used in the current study therefore necessarily included a broad range of medically recommended activities. For example, adherence could reflect making physician-recommended diet and exercise changes, which are common for fibromyalgia (Carville et al. 2008), and/or following medication regimens, which is common for managing and treating cancer and its symptoms (Naeim et al. 2008). Although the exact adherence behaviours engaged in could not be determined by the use of the general measure of adherence, we believe that the trade-off of fidelity for bandwidth that results from taking this approach is warranted as it provides foundational evidence to inform more focused studies into adherence behaviours.

### Limitations and Future Directions

Despite the novelty of our findings, they should be considered within the context of several limitations. Although adherence was measured with one of the most widely used self-report measures, the MOS-GA (Sherbourne et al. 1992), like all self-report measures it relies upon retrospective recall of adherence behaviours which are very dependent upon memory and recollection. Using objective measures of adherence such as prescription records and behavioural observations could provide more compelling support for the associations suggested by the current research. Nonetheless, a review of the literature on self-report measures of adherence found that questionnaires tend to have moderate-to-high concordance with other measures of medication adherence (Garber et al. 2004), suggesting that the current results are somewhat reliable. It may also be that self-compassion is more relevant for adherence to certain types of health recommendations, such as diet and exercise changes, which are known to be linked to self-compassion (Sirois et al. 2015a) and which have higher non-adherence rates compared to medication use among those with chronic health conditions (de Ridder et al. 2008).

The use of the short version of the self-compassion scale also limited our ability to more closely examine which specific components of self-compassion may be most beneficial for adherence behaviour, either via reducing stress or more directly by managing the negative affective states which can derail health behaviours. For example, it is possible that the mindfulness component with its focus on not over-identifying with the negative emotions after lapses may play a more central role in adherence because it helps diffuse the negative feelings which are known to threaten effective self-regulation (Sirois 2015b; Wagner and Heatherton 2015). Alternatively, self-kindness may be particularly beneficial for reducing the collateral stress from the self-judgements and self-blaming that can occur after a lapse in behaviour. Responding with acceptance and non-judgement about one’s medical condition may also be beneficial on its own, especially when the medical condition has some stigma attached to it (e.g. HIV, see Brion et al. 2014), as is the case with the fibromyalgia and chronic fatigue syndrome samples in our study.

The cross-sectional study design precludes drawing any firm conclusions about the presumed links between self-compassion and adherence. However, the direction of the relationships assumed in the current study are supported by theory (Friis et al. 2015a; Terry and Leary 2011), experimental research on stress (Arch et al. 2014; Breines et al. 2014), longitudinal research in a sample of people with celiac disease (Dowd and Jung 2017), self-compassion intervention research focused on weight management in healthy adults (Mantzios and Wilson 2015), and self-care in diabetes (Friis et al. 2016). Longitudinal and experimental research focusing on specific adherence behaviours across a wider range of chronic conditions would be useful to confirm the temporal precedence assumed in the current research.

Only four of the five samples used a version of the perceived stress scale (Cohen et al. 1983), with the fifth sample using the stress subscale of the DASS (Lovibond and Lovibond 1995). Nonetheless, the meta-analysis of the kappa-squared statistics, which captured the amount of variance in medical adherence explainable by self-compassion that could be attributed to perceived stress, suggested that there was not a significant amount of heterogeneity in the effects, despite the different measures of stress used. This lends some confidence to the findings and suggests that the effects noted are robust to different measures of perceived stress.

The type of physician recommendation that adherence was based upon was not recorded in the current study. Although previous research on the determinants of adherence found that adherence rates and the factors that predicted adherence varied widely across different health conditions (DiMatteo 2004), the test of heterogeneity in the current research indicated that the associations of self-compassion with adherence did not vary significantly across the five different medical samples. This may have to do with commonalities in the types of challenges faced by each of the medical groups tested in the current research. For example, cancer, fibromyalgia, and chronic fatigue have fatigue as a common and prominent symptom (Carlson et al. 2004; Clayton 2015; Wolfe et al. 2010). As fatigue is well-known to interfere with the self-regulation of health behaviours (e.g. Hagger et al. 2010), it may be that self-compassion is not as relevant for adherence in other chronic health conditions that do not have fatigue as a central symptom. Research with other chronic illnesses is therefore needed to better understand the extent to which self-compassion predicts different types of medical adherence among diverse medical groups.

Overall, our findings demonstrate that self-compassion is associated with better medical adherence among people with fibromyalgia, chronic fatigue syndrome, and cancer, due in part to lower stress. This research contributes to a growing evidence base indicating the value of self-compassion for health-related behaviours and outcomes in a variety of medical populations (Sirois and Rowse 2016). Further work is needed to gain a more nuanced perspective on how each of the components of self-compassion may be beneficial for adherence across different medical populations and doctor-recommended behaviours and to test other potential explanatory pathways beyond stress.
